# Escalated (Dependent) Oxycodone Self-Administration Is Associated with Cognitive Impairment and Transcriptional Evidence of Neurodegeneration in Human Immunodeficiency Virus (HIV) Transgenic Rats

**DOI:** 10.3390/v14040669

**Published:** 2022-03-24

**Authors:** Yu Fu, Irene Lorrai, Barry Zorman, Daniele Mercatelli, Chase Shankula, Jorge Marquez Gaytan, Celine Lefebvre, Giordano de Guglielmo, Hyunjae Ryan Kim, Pavel Sumazin, Federico M. Giorgi, Vez Repunte-Canonigo, Pietro Paolo Sanna

**Affiliations:** 1 Department of Immunology and Microbiology, The Scripps Research Institute, La Jolla, San Diego, CA 92037, USA; fuyu0413@gmail.com (Y.F.); ilorrai@scripps.edu (I.L.); cshankula@scripps.edu (C.S.); jmarquez@scripps.edu (J.M.G.); celinelef@gmail.com (C.L.); 2 European Bioinformatics Institute (EMBL-EBI), Hinxton CB10 1SD, UK; 3 Department of Pediatrics, Dan L. Duncan Cancer Center, Baylor College of Medicine, Houston, TX 77030, USA; barry.zorman@bcm.edu (B.Z.); ryan.kim2112@gmail.com (H.R.K.); pavel.sumazin@bcm.edu (P.S.); 4 Department of Pharmacy and Biotechnology, University of Bologna, 40126 Bologna, Italy; danielemercatelli@gmail.com (D.M.); federico.giorgi@unibo.it (F.M.G.); 5 92160 Antony, France; 6 Department of Psychiatry, University of California, La Jolla, San Diego, CA 92093, USA; gdeguglielmo@ucsd.edu

**Keywords:** neuroHIV, AIDS, neuroinflammation, cognitive impairment

## Abstract

Substance use disorder is associated with accelerated disease progression in people with human immunodeficiency virus (HIV; PWH). Problem opioid use, including high-dose opioid therapy, prescription drug misuse, and opioid abuse, is high and increasing in the PWH population. Oxycodone is a broadly prescribed opioid in both the general population and PWH. Here, we allowed HIV transgenic (Tg) rats and wildtype (WT) littermates to intravenously self-administer oxycodone under short-access (ShA) conditions, which led to moderate, stable, “recreational”-like levels of drug intake, or under long-access (LgA) conditions, which led to escalated (dependent) drug intake. HIV Tg rats with histories of oxycodone self-administration under LgA conditions exhibited significant impairment in memory performance in the novel object recognition (NOR) paradigm. RNA-sequencing expression profiling of the medial prefrontal cortex (mPFC) in HIV Tg rats that self-administered oxycodone under ShA conditions exhibited greater transcriptional evidence of inflammation than WT rats that self-administered oxycodone under the same conditions. HIV Tg rats that self-administered oxycodone under LgA conditions exhibited transcriptional evidence of an increase in neuronal injury and neurodegeneration compared with WT rats under the same conditions. Gene expression analysis indicated that glucocorticoid-dependent adaptations contributed to the gene expression effects of oxycodone self-administration. Overall, the present results indicate that a history of opioid intake promotes neuroinflammation and glucocorticoid dysregulation, and excessive opioid intake is associated with neurotoxicity and cognitive impairment in HIV Tg rats.

## 1. Introduction

Substance use disorder in people with human immunodeficiency virus (HIV; PWH) is associated with treatment non-compliance, an increase in viral transmission, and the clinical progression of HIV disease [1,2,3,4,5,6,7,8,9,10,11,12]. The nonmedical use of opioids has increased dramatically and is higher in North America than elsewhere in the world [13,14]. People with HIV have a higher prevalence of chronic pain [15,16,17] and are more likely to be prescribed opioids at higher doses and for longer periods of time than the general population [18,19,20,21]. Opioid use disorder (OUD) and problem opioid use, including high-dose opioid therapy and prescription drug misuse, are prevalent among PWH [22,23,24,25,26,27]. Oxycodone is among the most prescribed and misuse opioids in both the general population and PWH [15,28,29]. In a recent retrospective study, oxycodone accounted for the vast majority (71%) of 8744 opioid prescriptions in PWH [15]. In that study, 40% of opioid prescriptions were long-term (>365 days), and about half of them were chronic high-dose prescriptions [15].

Initial and occasional drug use is motivated by positive reinforcement [30]. The acute reinforcing effects of drugs of abuse are modeled by paradigms of short access (ShA) to drug self-administration [31]. In these models, rats are allowed to self-administer drugs of abuse for less than 3 h/day, producing stable levels and patterns of intake. Addiction is characterized by the loss of control in limiting intake and compulsion to take the drug [30], which can be modeled by paradigms of long access (LgA) to intravenous drug self-administration (12 h/day for opioids) [32,33,34,35,36], leading to escalated (dependent) drug intake [37]. This paradigm of escalated drug intake under LgA conditions is highly relevant to the human condition and has been suggested to model all seven criteria for drug addiction in the *Diagnostic and Statistical Manual of Mental Disorders*, 4th edition (DSM-IV), and seven of the 11 criteria for substance use disorder in the DSM-5 [37].

To model the effects of opioid misuse in HIV, we used HIV transgenic (Tg) rats that express multiple HIV products [38,39] and exhibit changes in gene expression that are consistent with human neuroHIV [40]. HIV Tg rats and wildtype (WT) littermates were tested for voluntary intravenous oxycodone self-administration under either ShA conditions (1 h/day), which is characterized by a nondependent “recreational”-like pattern of oxycodone use, and oxycodone self-administration under LgA conditions (12 h/day), which leads to escalated (dependent) oxycodone intake [32,41,42].

Here, we show that escalated oxycodone self-administration under LgA conditions induces cognitive impairment in HIV Tg rats. Impairments in medial prefrontal cortex (mPFC) function and frontostriatal connectivity are involved in the progression to compulsive drug intake and cognitive impairment in neuroHIV [43,44,45,46,47]. To better understand the molecular basis of detrimental interactions between HIV with excessive oxycodone intake, we profiled gene expression from the mPFC in HIV Tg and WT rats with a history of oxycodone self-administration under either ShA or LgA conditions and control littermate rats. Previous studies from our group showed that changes in gene expression that are associated with escalated cocaine, heroin, and alcohol self-administration are considerably different from changes in gene expression that are induced by a moderate “recreational”-like pattern of self-administration [31,36,48,49,50,51,52,53,54]. Gene expression analysis of the mPFC in HIV Tg rats that self-administered oxycodone under ShA conditions showed evidence of greater neuroinflammation than WT littermates that self-administered oxycodone under the same conditions. HIV Tg rats that escalated their oxycodone self-administration under LgA conditions exhibited transcriptional evidence of greater neuronal damage and neurodegeneration than WT littermates that self-administered oxycodone at comparable levels under the same conditions. Differential expression of the glucocorticoid-responsive genes Tsc22d3 (*Gilz*) and serum/glucocorticoid-regulated kinase 1 (*Sgk1*) indicated that glucocorticoid dysregulation and the neurotoxic actions of HIV products likely contribute to neurodegeneration and cognitive impairment in HIV Tg rats with a history of oxycodone self-administration.

Altogether, the present results indicate that voluntary oxycodone intake and HIV result in an increase in neuroinflammation in the mPFC in rats with a history of nondependent oxycodone self-administration under ShA conditions and neurotoxicity and neurodegeneration in rats with a history of dependent oxycodone self-administration under LgA conditions.

## 2. Materials and Methods

### 2.1. Animals

Male HIV Tg rats (*n* = 27) and WT littermate control rats (*n* = 28) that were backcrossed on a Wistar background were housed two per cage on a reverse 12 h/12 h light/dark cycle (lights off at 8:00 a.m.) in a temperature (20–22 °C) and humidity (45–55%) controlled vivarium with *ad libitum* access to tap water and food pellets (PJ Noyes, Lancaster, NH, USA). All of the procedures were conducted in strict adherence to the National Institutes of Health *Guide for the Care and Use of Laboratory Animals* and were approved by the Institutional Animal Care and Use Committee of The Scripps Research Institute. At the time of testing, the rats’ body weights ranged between 350 and 400 g.

### 2.2. Intravenous Catheterization

The animals were anesthetized by the inhalation of a mixture of isoflurane/oxygen, and intravenous catheters were aseptically inserted in the right jugular vein using a modified version of a procedure that was described previously [55,56]. The vein was punctured with a 22-gauge needle, and the tubing was inserted and secured inside the vein by tying the vein with suture thread. The catheter assembly consisted of an 18-cm length of Micro-Renathane tubing (0.023-inch inner diameter, 0.037-inch outer diameter; Braintree Scientific, Braintree, MA, USA) that was attached to a guide cannula (Plastics One, Roanoke, VA, USA). The guide cannula was bent at a near right angle, embedded in dental acrylic, and anchored with 2-cm square mesh. The catheter exited through a small incision on the back, and the base was sealed with a small plastic cap and metal cover cap. This design helped keep the catheter base sterile and protected. The catheters were flushed daily with heparinized saline (10 U/mL of heparin sodium; American Pharmaceutical Partners, Schaumburg, IL, USA) in 0.9% bacteriostatic sodium chloride (Hospira, Lake Forest, IL, USA) that contained 20 mg/0.2 mL of the antibiotic Timentin (GlaxoSmithKline, Middlesex, UK).

### 2.3. Drugs

Oxycodone HCl (National Institute on Drug Abuse, Bethesda, MD, USA) was dissolved in 0.9% saline (Hospira, Lake Forest, IL, USA) and self-administered intravenously at a dose of 0.15 mg/kg/infusion [33].

### 2.4. Oxycodone Self-Administration

Self-administration sessions were performed in operant conditioning chambers (Med Associates, St. Albans, VT, USA) that were enclosed in lit, sound-attenuating, ventilated environmental cubicles. The back wall of each operant chamber was illuminated by a white house light. The front panel had two retractable response levers and two cue lights above them. At the beginning of each self-administration session, the white house light was on, and the two levers were extended. Responses on the right (active) lever resulted in the delivery of 0.1 mL of oxycodone solution by the activation of an infusion pump that was outside the operant chamber. A 20 s timeout (TO) period, signaled by illumination of the cue light above the active lever, was interposed between each active lever response to avoid possible oxycodone overdose. Responses on the left (inactive) lever were recorded but did not have any scheduled consequences. Fluid delivery and responses on both levers were controlled and recorded by a computer that interfaced with each operant chamber. In the present study, the rats had access to oxycodone under a fixed-ratio 1 (FR1) schedule of reinforcement. Oxycodone was delivered at 0.15 mg/kg/0.1 mL.

After 1 week of recovery from intravenous catheterization surgery, the rats were trained to lever-press for oxycodone over 10 consecutive 1-h self-administration sessions. The rats were then allowed to self-administer oxycodone in 1 h (ShA group) or 12 h (LgA group) sessions for 21 consecutive days. The animals were then subjected to periods of forced abstinence, followed by the resumption of oxycodone self-administration under the same conditions.

### 2.5. Novel Object Recognition Test

The novel object recognition (NOR) test was conducted on two consecutive days in a black square arena (60 × 60 cm). On Day 1 (habituation), the rats were individually placed in the empty arena and allowed to freely explore it for 5 min. The next day (Day 2, training), two identical objects (A and A’) were placed in the arena, and the rat was allowed to freely interact with both objects for 10 min. On the same day, 1 h after training (Day 2, test), one of the familiar objects was changed to a novel object (A and B). The Recognition Index (RI), defined as the ratio between the time spent with the novel object/time spent with both objects [novel + familiar] × 100, was calculated over the 5 min test. The arena and objects were cleaned with a 70% alcohol solution before each rat underwent the NOR test.

### 2.6. Total RNA Isolation and RNA-Sequencing

**Figure 1:**
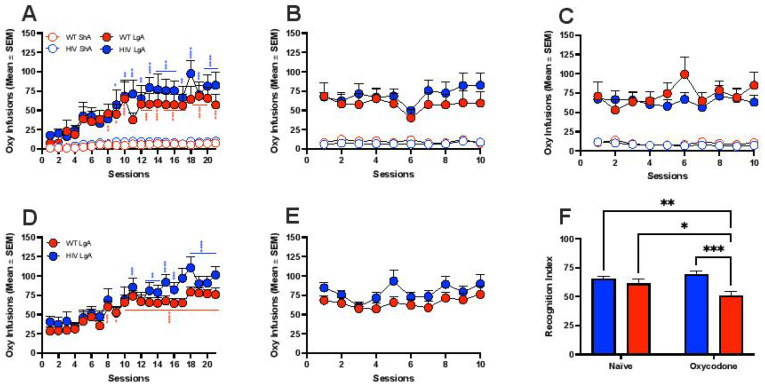
Intravenous oxycodone self-administration in HIV Tg rats and WT rats under nondependent (short access (ShA)) and dependent (long access (LgA)) conditions. (**A**) HIV Tg rats and WT littermates self-administering oxycodone under a fixed-ratio 1 (FR1) schedule under short-access (ShA) conditions in 1-h daily sessions or under long-access (LgA) conditions in 12-h daily sessions. HIV Tg rats and WT littermates escalated oxycodone self-administration under LgA conditions. Oxycodone intake did not differ between HIV Tg and WT rats under either ShA or LgA conditions. * *p* < 0.05, ** *p* < 0.005, *** *p* < 0.0005, **** *p* < 0.0001 vs. Session 1 (Newman–Keuls *post hoc* test). (**B**) Following a period of enforced abstinence to model the intermittent pattern of opioid abuse in humans, HIV Tg and WT rats both under ShA and LgA conditions promptly resumed their previous levels of self-administration, which did not differ between genotypes. (**C**) Oxycodone self-administration under ShA and LgA conditions did not differ between genotypes after a second period of enforced abstinence. (**D**) The pattern of oxycodone self-administration in HIV Tg and WT rats under LgA conditions was highly reproducible and closely replicated in an independent set of rats. * *p* < 0.05, ** *p* < 0.005, *** *p* < 0.0005, **** *p* < 0.0001 vs. Session 1 (Newman–Keuls *post hoc* test). (**E**) Following a period of enforced abstinence, rats of both genotypes promptly resumed their previous levels of self-administration, which did not differ between genotypes. (**F**) HIV Tg rats with a history of escalated oxycodone self-administration under LgA conditions performed significantly worse than WT littermates in the NOR task during protracted withdrawal. HIV Tg rats vs. naive HIV Tg rats: * *p* < 0.05; HIV Tg rats vs. naive WT rats: * *p* < 0.05; HIV Tg rats vs. WT rats: ** *p* < 0.005; Newman–Keuls *post hoc* test.

HIV Tg and WT rats (Figure 1A–C) were sacrificed 48 h after last self-administration session. Microdissected tissue from the mPFC was processed for total RNA isolation using the *mir*Vana miRNA Isolation Kit (ThermoFisher Scientific, Waltham, MA, USA) and Zymo Purification Kit (Zymo Research, Irvine, CA, USA). Libraries were prepared with the KAPA mRNA HyperPrep Kit for Illumina sequencing (KAPABiosystems, Wilmington, MA, USA) for mRNA capture with magnetic oligo-dT beads, cDNA synthesis, and library construction and amplification. The Poly-A libraries were subsequently sequenced on an Illumina HiSeq4000 sequencer at 30-million-read target coverage (100 bp paired-end reads).

### 2.7. Gene Expression Profiling and Gene Set Enrichment Analysis

The sequences were first trimmed using Trimmomatic with default setting (version 0.39). All samples passed the quality control by fastQC (version 0.11.9). Fastq files were aligned to combined rat (RatBN7.2) and HIV-1 (NC_001802) using Bowtie2 [57] with default settings. Transcript expression was normalized using RSEM (version 1.3.0) [58]. The two transcriptomes were combined as described previously [59]. The rat genome was humanized using the biomaRt package from R software. Differential expression analysis was performed using the DESeq2 package in R software (the Wald method was used). Gene Set Enrichment Analysis (GSEA) [60] was performed in R software for MSigDB-curated gene sets but excluding perturbation-based gene sets for a total of 1452 MSigDB gene sets. Multiple testing adjustment was performed using the False Discovery Rate. Fastq files were deposited in the European Nucleotide Archive project (PRJEB49963).

### 2.8. Quantitative Polymerase Chain Reaction Validation

Differentially expressed genes in the mPFC were validated in HIV and WT rats with or without a history of oxycodone exposure using the SYBR Green fluorescence detection kit with the CFX96 Touch real-time PCR detection system (Bio-Rad, Hercules, CA, USA). A set of optimized real-time polymerase chain reaction (RT-PCR) primer assays was designed, and the following sequences were used: TSC22D3 (GGC CCT AGA CAA GAT TGA [sense] and GCT CAC GAA TCT GCT CCT TTA [antisense]) and β-actin (AGATTACTGCCCTGGCTCCT [sense] and CAGTGAGGCCAGGATAGAGC [antisense]). Gene expression was normalized to β-actin and analyzed based on the ^ΔΔ^CT method.

## 3. Results

### 3.1. Oxycodone Self-Administration in HIV Transgenic Rats

HIV Tg rats and WT littermates were allowed to intravenously self-administer oxycodone under an FR1 schedule whereby one lever press resulted in one oxycodone injection under ShA conditions (1-h daily sessions) or under LgA conditions (12-h daily sessions; Figure 1A). HIV Tg and WT rats exhibited similar patterns of the acquisition of self-administration and oxycodone intake under both ShA (two-way repeated-measures analysis of variance [ANOVA]; genotype: *F*_1,13_ = 4.72, *p* > 0.05; session: *F*_20,260_ = 13.73, *p* < 0.0001; interaction: *F*_20,260_ = 1.060, *p* > 0.05) and LgA conditions (two-way repeated-measures ANOVA; genotype: *F*_1,13_ = 0.4767, *p* > 0.05; session: *F*_20,260_ = 13.36, *p* < 0.0001; interaction: *F*_20,260_ = 0.988, *p* > 0.05). Moreover, as expected, HIV Tg and WT rats progressively escalated their oxycodone intake over the 21 consecutive sessions of LgA (Figure 1A). After 21 sessions of oxycodone self-administration, HIV Tg and WT rats with histories of either ShA or LgA self-administration underwent a period of enforced abstinence to model the intermittent pattern of opioid misuse in humans, in which oxycodone was unavailable for 2 weeks. Following the restoration of access to self-administration, HIV Tg and WT rats under both ShA and LgA conditions promptly resumed their previous levels of self-administration, which did not differ between genotypes (two-way repeated-measure ANOVA for ShA: genotype: *F*_1,13_ = 2.013, *p* > 0.05; session: *F*_9,117_ = 1.525, *p* > 0.05; interaction: *F*_9,117_ = 0.839, *p* > 0.05; two-way repeated-measure ANOVA for LgA: genotype: *F*_1,13_ = 0.445, *p* > 0.05; session: *F*_9,117_ = 4.25, *p* < 0.0001; interaction: *F*_9,117_ = 1.339, *p* > 0.05; Figure 1B). Similar results were observed after a second period of 2 weeks of enforced abstinence (two-way repeated-measure ANOVA for ShA: genotype: *F*_1,12_ = 0.874, *p* > 0.05; session: *F*_9,108_ = 3.309, *p* > 0.05; interaction: *F*_9,108_ = 0.807, *p* > 0.05; two-way repeated-measures ANOVA for LgA: genotype: *F*_1,13_ = 0.257, *p* > 0.05; session: *F*_9,117_ = 2.219, *p* < 0.05; interaction: *F*_9,117_ = 1.754, *p* > 0.05; Figure 1C). Oxycodone self-administration was replicated in an independent set of HIV Tg and WT rats (Figure 1D). The statistical analysis indicated that both groups escalated their oxycodone intake when exposed to 21 consecutive sessions of self-administration (Figure 1), and no difference was found between genotypes (two-way repeated-measures ANOVA: genotype: *F*_1,23_ = 1.47, *p* > 0.05; session: *F*_20,460_ = 21.40, *p* < 0.0001; interaction: *F*_20,460_ = 1.13, *p* > 0.05; Figure 1D). Subsequently, after 4 weeks of forced abstinence, the rats were re-allowed to self-administer oxycodone, again confirming the lack of difference in drug intake over 10 consecutive sessions of LgA (two-way repeated-measures ANOVA: genotype: *F*_1,23_ = 0.3275, *p* > 0.05; session: *F*_9,207_ = 6.18, *p* < 0.0001; interaction: *F*_9,207_ = 0.721, *p* > 0.05; Figure 1E).

To investigate the cognitive consequences of a history of escalated (dependent) oxycodone self-administration in HIV Tg rats, we tested the rats in the NOR task [61,62] in protracted withdrawal (2 weeks) during the period of enforced abstinence after the initial 21 sessions of self-administration under LgA conditions (Figure 1F). The NOR paradigm was performed with a 1 h delay after exposure to the familiar objects. The two-way ANOVA indicated a significant effect of genotype (*F*_1,42_ = 11.25, *p* < 0.005) and a significant genotype × treatment interaction (*F*_1,42_ = 4.338, *p* < 0.05) but no effect of treatment (*F*_1,42_ = 0.878, *p* > 0.05). A history of escalated oxycodone self-administration did not affect the RI in WT rats. Conversely, HIV Tg rats had a lower RI compared with naive HIV Tg, naive WT rats, and WT rats with a history of oxycodone self-administration (Figure 1F).

These data indicate that a history of escalated (dependent) oxycodone self-administration is associated with impairments in working memory in the NOR paradigm in HIV Tg rats but not in WT rats, despite their comparable levels of oxycodone intake.

### 3.2. Gene Expression Profiling in the mPFC in HIV Tg and WT Rats That Self-Administered Oxycodone under Nondependent (ShA) and Dependent (LgA) Conditions

**Figure 2:**
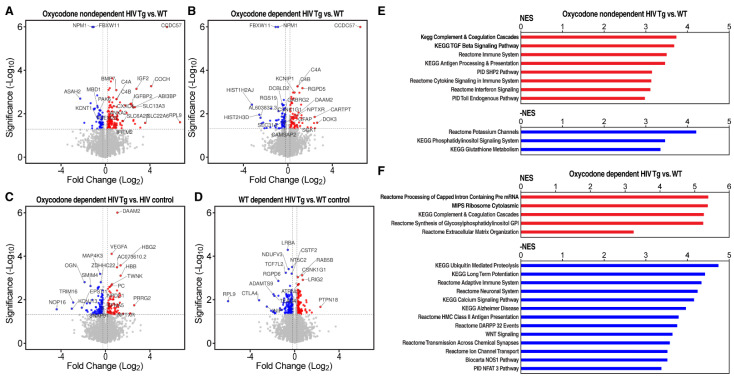
Gene expression profiling in the mPFC in HIV Tg and WT rats that self-administered oxycodone under nondependent (ShA) and dependent (LgA) conditions. (**A**) Volcano plot of changes in gene expression in the mPFC in HIV Tg rats with a history of oxycodone self-administration under ShA conditions compared with oxycodone-self-administering WT rats under the same conditions. The plots show significance (Log10 of *p* value) vs. fold-change (Log2) on the y and x axes, respectively. Genes that significantly increased in HIV Tg rats vs. WT rats with oxycodone self-administration under ShA conditions are indicated in red. Genes that significantly decreased are indicated in blue. (**B**) Volcano plot of changes in gene expression in the mPFC in HIV Tg rats with a history of oxycodone self-administration under LgA conditions compared with oxycodone-self-administering WT rats under the same conditions. Genes that significantly increased in HIV Tg rats vs. WT rats that self-administered oxycodone under LgA conditions are indicated in red. Genes that significantly decreased are indicated in blue. (**C**) Volcano plot of changes in gene expression in the mPFC in HIV Tg rats with a history of oxycodone self-administration under LgA conditions compared with oxycodone-naive HIV Tg rats and (**D**) volcano plot of changes in gene expression in the mPFC in WT rats with a history of oxycodone self-administration under LgA conditions compared with oxycodone-naive WT rats. (**E**) Pathway analysis by GSEA [60] of HIV Tg rats vs. WT rats that self-administered oxycodone under ShA conditions. Transcriptional evidence of an increase in neuroinflammation was seen in HIV Tg rats compared with WT rats that self-administered oxycodone under the same conditions. (**F**) Pathway analysis of HIV Tg rats vs. WT rats that self-administered oxycodone under LgA conditions. Transcriptional evidence of an increase in neuronal injury and neurodegeneration was seen in HIV Tg rats compared with WT rats under the same conditions. NES, normalized enrichment score [60].

Genes that significantly increased in HIV Tg rats vs. WT rats that self-administered oxycodone under ShA conditions included complement component 4A and B (*C4a*, *C4b*; which is implicated in neuroinflammation and Alzheimer’s disease) [63,64], annexin A2 (*Anxa2*; a proinflammatory factor [65] that has been implicated in immune-mediated diseases and viral infections) [66,67], the transforming growth factor β (TGF-β) family member *Bmp7*, interferon-induced transmembrane protein 2 (*Ifitm2*), CXXC finger protein 4 (*Cxxc4/Idax*; a negative regulator of WNT signaling and epigenetic regulator), *Igf2* (a mitogenic and neuroprotective peptide that is associated with inflammation in different settings) [68,69], insulin-like growth factor-binding protein 2 (*Igfbp2*), and *Slc6a20* (a regulator of brain glycine and *N*-methyl-D-aspartate [NMDA] receptor function) [70]; Figure 2A–D, Supplemental Tables S1–S6).

**Figure 3:**
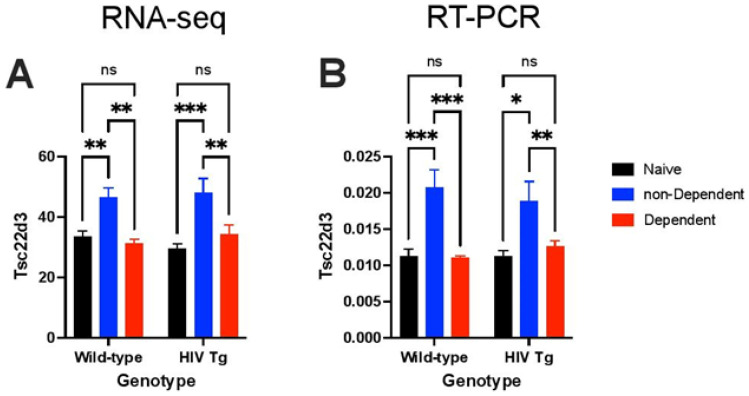
Differential expression of the glucocorticoid-responsive gene *Tsc22d3* (*Gilz*) by histories of nondependent (ShA) and dependent (LgA) oxycodone self-administration in HIV Tg rats and WT rats. (**A**) RNA-sequencing expression of the glucocorticoid-responsive gene *Tsc22d3* (*Gilz*) increased in both HIV Tg rats and WT rats with histories of oxycodone self-administration under ShA conditions compared with their respective oxycodone-naive controls, which was indicative of dysregulation of glucocorticoid-dependent gene expression associated with oxycodone self-administration. *Tsc22d3* expression did not increase in HIV Tg rats or WT rats with histories of oxycodone self-administration under LgA conditions, consistent with adaptations that occur under conditions of chronic elevations of glucocorticoids [82,83] (*F*_2,34_ = 21.91, *p* < 0.0001, *n* = 6–7/group). (**B**) Consistent results were obtained by RT-PCR (*F*_2,36_ = 19.64, *p* < 0.0001, *n* = 7–8 /group). * *p* < 0.05, ** *p* < 0.01, *** *p* < 0.001 (Newman–Keuls *post hoc* test).

The glucocorticoid-responsive gene *Tsc22d3* (glucocorticoid-induced leucine zipper [*Gilz*]) [71] was increased in both HIV Tg rats and WT rats with histories of oxycodone self-administration under ShA conditions compared with their respective oxycodone-naive controls (Figure 3).

Genes that significantly decreased in HIV Tg rats vs. WT rats that self-administered oxycodone under ShA conditions included the epigenetic factor *Mbd1*, *Pak6* (a member of the group B family of PAK serine/threonine kinases), *Kcnt1* (which encodes a sodium-gated potassium channel that is implicated in cellular excitability and seizures) [72], and *Tmem25* (a regulator of NMDA receptor function and excitability) [73].

Genes that significantly increased in HIV Tg rats vs. WT rats that escalated their oxycodone self-administration under LgA conditions included potassium voltage-gated channel interacting protein 1 (*Kcnip1*/*Kchip1*) [74], *Daam2* (which encodes a protein that contributes to Wnt signaling and regenerative myelination) [75], and glial fibrillary acidic protein (*Gfap*; which is indicative of astrogliosis).

Genes that significantly decreased in HIV Tg rats vs. WT rats that escalated their oxycodone self-administration under LgA conditions included F-Box and WD repeat domain containing 11 (*Fbxw11*; also known as β-transducin repeat containing protein 2 [βTrCP2]), homolog of Slimb (*Hos*)), *Rgs19* (G-α-interacting protein [*Gaip*]; a modulator of dopaminergic signaling) [76], the γ-1 isoform of casein kinase 1 (*Csnk1g1*; which is associated with syndromic developmental delay and autism spectrum disorder) [77], *Pcdh19* (the causal gene of a form of clustering epilepsy [PCDH19-CE]) [78], the novel scaffolding receptor *Dcbld2* [79], *Sec31a* (which was recently identified as an ortholog of the *Drosophila* gene by the same name, the null mutation of which was shown to cause a severe neurological syndrome) [80], the γ-aminobutyric acid receptor subunit γ-2 (*Gabrg2*), neurofilament medium polypeptide (NF-M), microtubule minus-end binding protein (*Camsap2*; which controls axon and dendrite morphogenesis) [81], and the neuronal pentraxin receptor (*Nptxr*).

*Sgk1*, a glucocorticoid-responsive gene that has been implicated in Alzheimer’s disease and Parkinson’s disease [84,85], was elevated in HIV Tg rats vs. WT rats that escalated their oxycodone self-administration under LgA conditions (Figure 1B). *Sgk1* was also elevated in both HIV and WT rats that self-administered oxycodone under ShA conditions compared with their respective oxycodone-naive control littermates (Supplemental Tables). *Tsc22d3* (*GILZ*) was not differentially regulated in HIV Tg rats and WT rats with histories of oxycodone self-administration under LgA conditions (Figure 3), presumably indicating an adaptation to chronic glucocorticoid dysregulation, consistent with conditions that are characterized by chronically elevated glucocorticoids [82,83]. Differential regulation of the glucocorticoid-responsive genes *Tsc22d3* and *Sgk1* suggests a role for glucocorticoids in changes in gene expression that are caused by a history of oxycodone self-administration.

### 3.3. Transcriptional Evidence of Increases in Neuroinflammation, Neuronal Injury, and Neurodegeneration in HIV Tg Rats with a History of Oxycodone Self-Administration

**Figure 4:**
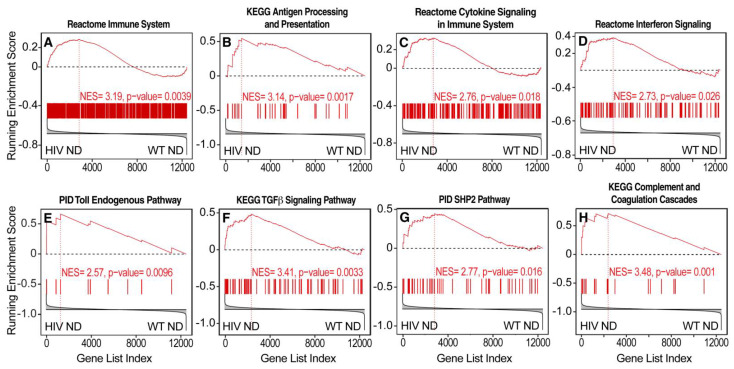
Transcriptional evidence of an increase in neuroinflammation in HIV Tg rats that self-administered oxycodone under nondependent (short access (ShA)) conditions. Pathway analysis by GSEA provided evidence of (**A**,**B**) broad immune activation and (**C**) the induction of cytokine signaling, including (**D**) interferon (IFN) signaling, (**E**) Toll signaling, (**F**) TGFβ, (**G**) SHP2 signaling, and (**H**) complement and coagulation cascades. Each bar represents a gene in the gene set [60]. HIV ND, HIV Tg rats that self-administered oxycodone under nondependent (ShA) conditions; WT ND, wildtype rats that self-administered oxycodone under nondependent conditions. NES = normalized enrichment score [60].

**Figure 5:**
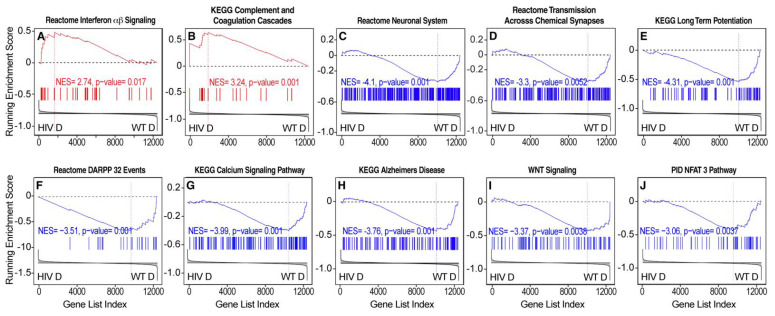
Transcriptional evidence of increases in neuronal injury and neurodegeneration in HIV Tg rats that self-administered oxycodone under dependent (LgA) conditions. Representative pathways that were differentially activated by GSEA are indicative of an increase in the expression of (**A**) interferon (IFN) signaling and (**B**) complement (which has been implicated in increases in inflammation and neurodegeneration [88]) and the broad downregulation of (**C**) neuronal genes, including genes that are involved in (**D**) neuronal communication, (**E**) neural plasticity, and (**F**,**G**) signaling and (**H**) genes that are involved in neurodegenerative conditions (e.g., Alzheimer’s disease) and (**I**,**J**) trophism. Each bar represents a gene in the gene set [60]. NES, normalized enrichment score [60]; HIV D, HIV Tg rats that self-administered oxycodone under dependent (long access (LgA)) conditions; WT D, wildtype rats that self-administered oxycodone under dependent conditions. NES = normalized enrichment score [60].

**Figure 6:**
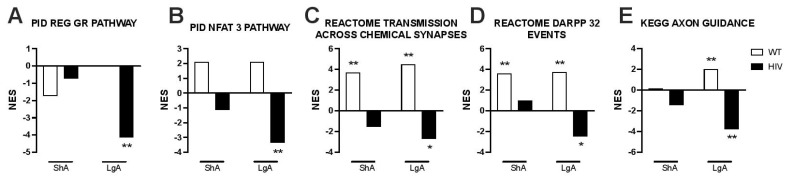
Differential regulation of selected pathways by histories of nondependent (ShA) and dependent (LgA) oxycodone self-administration in HIV Tg rats and WT rats. The figure shows the differential regulation of gene sets that are relevant to neurodegeneration in HIV Tg rats and WT rats with histories of nondependent (ShA) and dependent (LgA) oxycodone self-administration. (**A**) Glucocorticoid regulated genes showed greater adaptations in HIV Tg rats with a history of dependent (LgA) oxycodone self-administration. (**B**) Nfat3 regulated genes, (**C**) genes that are involved in neuronal communication, including synaptodendritic genes, (**D**) genes that are related to signaling, such as DARPP32 regulated events, and (**E**) genes that are related to axonal function were downregulated in HIV Tg rats with a history of dependent (LgA) oxycodone self-administration. * *p* < 0.05, ** *p* < 0.01, vs. oxycodone-naive control rats of the respective genotype. NES = normalized enrichment score [60].

Pathway analysis was conducted by GSEA [60]. This method determines whether a gene set shows a significant concordant expression difference between two conditions, demonstrated by asymmetric distribution toward one of the two experimental conditions of the running enrichment score plot [60]. Pathways that are indicative of general immune activation, inflammation, and greater cytokine signaling were differentially activated in HIV Tg rats vs. WT rats that self-administered oxycodone under ShA conditions (Figure 2E and Figure 4). GSEA demonstrated differences in the regulation of pathways that are involved in neurodegeneration between HIV Tg rats and WT rats that self-administered oxycodone under LgA conditions, suggesting the differential activation of pathogenic mechanisms (Figure 2F, Figure 5 and Figure 6). Glucocorticoid-regulated genes showed greater adaptations in HIV Tg rats with a history of dependent (LgA) oxycodone self-administration (Figure 6A). We also observed the broad downregulation of neurodegeneration-related genes, including genes that are regulated by Nfat3, which is implicated in neuronal survival [86,87], synaptodendritic genes, and other genes that are involved in neuronal communication and neural plasticity (Figure 6B–E).

## 4. Discussion

Opioid use disorder has been shown to be associated with impairments in various cognitive domains, including working memory, executive function, and impulsivity [89,90]. Clinical evidence indicates that opioid misuse can promote cognitive impairment in PWH [91,92,93,94]. In the CNS HIV Antiretroviral Therapy Effects Research (CHARTER) study, lifetime heroin use was associated with worse recall and working memory [94].

Oxycodone and hydrocodone are the most prescribed Schedule II opioids [95,96]. Oxycodone and hydrocodone are powerful painkillers and among the most widely misused prescription drugs [95,96,97,98]. Oxycodone is among the most prescribed opioids in PWH [15].

Here, we found that HIV Tg rats that self-administered oxycodone under LgA conditions that led to escalated (dependent) drug intake exhibited significant impairments in working memory performance in the NOR paradigm compared with WT rats that self-administered oxycodone under the same conditions. However, oxycodone had comparable reinforcing potential in HIV Tg and WT rats, unlike methamphetamine self-administration, whereby HIV Tg rats exhibited an increase in methamphetamine intake under LgA conditions [99].

Working memory is the most commonly affected cognitive executive function among PWH [100]. The NOR paradigm is an established working memory paradigm that is sensitive to impairments in brain regions that are involved in memory, including the hippocampus and entorhinal, perirhinal, parahippocampal, and prefrontal cortices, among others [61,62]. The NOR paradigm does not involve rewards; instead, animals explore the novel object as part of their natural propensity toward novelty [61]. The NOR paradigm has been used to study working memory deficits that are induced by HIV products, such as Tat [101,102], and working memory deficits in HIV Tg rats [103].

The mPFC in rodents is a key region in working memory and cognitive flexibility [104,105,106]. Here, we found that gene expression profiling of the mPFC showed transcriptional evidence of an increase in inflammation in HIV Tg rats that self-administered oxycodone under ShA conditions compared with WT rats that self-administered oxycodone under the same conditions. In HIV Tg rats that self-administered oxycodone under LgA conditions, gene expression profiling showed transcriptional evidence of an increase in neuronal injury compared with WT rats that self-administered oxycodone under the same conditions.

Opioids have complex actions on inflammation and immune system activation. Morphine exposure has been shown to amplify microglial activation by lipopolysaccharide [107,108]. Morphine exposure exacerbates Tat-induced microglial activation in vitro [109] and in vivo [110] following short-term exposure. Morphine potentiates the release of cytokines by microglia and other cells that are exposed in vitro to lipopolysaccharide [107,108,111,112] or Tat [109]. The morphine-induced potentiation of cytokine production has been shown to be dose-dependent, and it was reduced at higher morphine concentrations [111,112]. The latter is consistent with the dose-dependent immunosuppressive actions of opioids [113,114].

We found that mRNA expression of the glucocorticoid responsive gene *Tsc22d3* (*Gilz*) increased in nondependent HIV Tg rats and WT rats under ShA conditions but was not different from control values in both oxycodone-dependent HIV Tg and WT rats under LgA conditions. *Tsc22d3* is induced by glucocorticoids [71]. However, brain *Tsc22d3* is not increased in conditions that are associated with chronic glucocorticoid activation, such as chronic stress [82] and major depressive disorder [83]. *Tsc22d3* contributes to the anti-inflammatory effects of glucocorticoids by inhibiting key proinflammatory transcription factors, such as nuclear factor-κB and adaptor protein-1, and modulates macrophage polarization [71,115,116,117,118,119]. Lower *Tsc22d3* and inflammatory gene expression in oxycodone-dependent rats in the present study may be a glucocorticoid-related adaptive response in rats with escalated oxycodone intake that are exposed to high levels of the drug. Consistent with this view, the glucocorticoid-responsive gene *Sgk1* was elevated in both HIV Tg and WT rats that self-administered oxycodone under both LgA and ShA conditions compared with their respective oxycodone-naive control littermates.

Cognitive impairment and evidence of an increase in neuronal injury in HIV Tg rats that self-administered oxycodone under LgA conditions may result from the interaction between HIV products and high doses of opioids and glucocorticoid dysregulation. Elevated glucocorticoid levels were also seen in Tat Tg mice that were exposed to oxycodone [120]. An increase in neuronal toxicity by exposure to morphine and Tat has been shown in in vitro model systems [121,122]. Moreover, opioid misuse is associated with cognitive impairments in the general population [89,90] and PWH [91,92,93,94]. Elevated plasma glucocorticoids are also associated with impairments in working memory in humans [123,124,125]. Prolonged hypercortisolemia induces mPFC and hippocampal impairments [126,127,128,129,130,131,132,133,134,135] and memory deficits [136,137,138]. Elevated cortisol levels are seen in neurodegenerative conditions, including Alzheimer’s disease, Parkinson’s disease, and Huntington’s disease, suggesting a general role for chronic activation of the hypothalamic–pituitary–adrenal (HPA) axis in neurodegeneration [139,140,141,142,143]. In human Alzheimer’s disease, plasma cortisol levels correlate with the degree of cognitive impairment, suggesting that HPA axis hyperactivity contributes to the progression of cognitive decline [142,144,145]. The glucocorticoid-responsive gene *SGK1* has been implicated in Alzheimer’s disease and Parkinson’s disease [84,85]. Aging is also associated with an increase in cortisol [146]. Rodents that are exposed to chronic stress exhibit reductions of glutamate receptor expression, reductions of markers of synaptic plasticity, and the atrophy of pyramidal cell dendrites in the mPFC [106,147,148,149].

Thus, an increase in inflammation in HIV Tg rats vs. WT rats that self-administered oxycodone under ShA conditions likely resulted from the combination of proinflammatory actions of HIV products with proinflammatory actions of opioids at lower levels of exposure [111,112]. In rats with escalated oxycodone intake under LgA conditions, the immunosuppressive [113,114] and neurotoxic [121,122] effects of high doses of opioids may predominate and be additive with the neurotoxic [123,124,125,126,127,128,129,130,131,132,133,134,135,136,137,138,139,140,141,142,143,144,145] and immunosuppressive [150] actions of glucocorticoids.

In conclusion, we provide transcriptional evidence of an increase in immune activation and neuroinflammation in HIV Tg rats vs. WT littermate control rats with histories of oxycodone self-administration under limited access (ShA) conditions, which leads to a moderate, stable, “recreational”-like level of oxycodone intake. In HIV Tg rats with histories of oxycodone self-administration under conditions of extended (LgA) access to self-administration, which led to considerably higher levels of oxycodone intake, we found transcriptional evidence of an increase in neuronal injury and neurodegeneration and significant impairments in memory performance in the NOR paradigm compared with WT rats that self-administered oxycodone under the same conditions. Transcriptional evidence of glucocorticoid dysregulation was seen in both HIV Tg rats and WT rats that self-administered oxycodone. The neurotoxic actions of HIV products, together with glucocorticoid-dependent adaptations, likely contribute to cognitive impairments in oxycodone-dependent HIV Tg rats.

## Data Availability

Fastq files were deposited in the European Nucleotide Archive project PRJEB49963.

## Supplementary Materials

The following supporting information can be downloaded at: https://www.mdpi.com/article/10.3390/v14040669/s1: Supplemental Table S1: Differential expression of oxycodone-dependent HIV Tg rats vs. WT rats (DE_HIV_D_WT_D), Supplemental Table S2: Differential expression of oxycodone nondependent HIV Tg rats vs. WT rats (DE_HIV_ND_WT_ND), Supplemental Table S3: Differential expression of oxycodone nondependent WT rats vs. oxycodone-naive WT rats (DE_WT_ND_WT_N), Supplemental Table S4: Differential expression of oxycodone nondependent (ND) HIV Tg rats vs. oxycodone-naive (N) HIV Tg rats (DE_HIV_ND_HIV_N), Supplemental Table S5: Differential expression of oxycodone-dependent WT rats vs. oxycodone-naive WT rats (DE_WT_D_WT_N), Supplemental Table S6: Differential expression of oxycodone-dependent HIV Tg rats vs. oxycodone-naive HIV Tg rats (DE_HIV_D_HIV_N).
